# Virtually impossible: limiting Australian children and adolescents daily screen based media use

**DOI:** 10.1186/1471-2458-15-5

**Published:** 2015-01-22

**Authors:** Stephen Houghton, Simon C Hunter, Michael Rosenberg, Lisa Wood, Corinne Zadow, Karen Martin, Trevor Shilton

**Affiliations:** Graduate School of Education, The University of Western Australia, Crawley, Perth, 6009 WA Australia; School of Psychological Sciences and Health, University of Strathclyde, Glasgow, Scotland; Health Promotion Evaluation Unit, The University of Western Australia, Crawley, Perth, Australia; School of Population Health, The University of Western Australia, Crawley, Perth, Australia; National Heart Foundation, The University of Western Australia, Crawley, Perth, Australia

**Keywords:** Screen use, Australia, Children, Adolescents, Screen activities, Guidelines

## Abstract

**Background:**

Paediatric recommendations to limit children’s and adolescents’ screen based media use (SBMU) to less than two hours per day appear to have gone unheeded. Given the associated adverse physical and mental health outcomes of SBMU it is understandable that concern is growing worldwide. However, because the majority of studies measuring SBMU have focused on TV viewing, computer use, video game playing, or a combination of these the true extent of total SBMU (including non-sedentary hand held devices) and time spent on specific screen activities remains relatively unknown. This study assesses the amount of time Australian children and adolescents spend on all types of screens and specific screen activities.

**Methods:**

We administered an online instrument specifically developed to gather data on all types of SBMU and SBMU activities to 2,620 (1373 males and 1247 females) 8 to 16 year olds from 25 Australian government and non-government primary and secondary schools.

**Results:**

We found that 45% of 8 year olds to 80% of 16 year olds exceeded the recommended < 2 hours per day for SBMU. A series of hierarchical linear models demonstrated different relationships between the degree to which total SBMU and SBMU on specific activities (TV viewing, Gaming, Social Networking, and Web Use) exceeded the < 2 hours recommendation in relation to sex and age.

**Conclusions:**

Current paediatric recommendations pertaining to SBMU may no longer be tenable because screen based media are central in the everyday lives of children and adolescents. In any reappraisal of SBMU exposure times, researchers, educators and health professionals need to take cognizance of the extent to which SBMU differs across specific screen activity, sex, and age.

## Background

Changes in children’s and adolescents’ lifestyles over several decades, particularly in relation to increased screen based media use (SBMU), sedentary behaviour and the associated adverse health outcomes [1–3] led The American Academy of Pediatrics AAP: [4] to recommend that children under two years of age have no screen exposure and that parents of children older than two years limit their children’s exposure to less than two hours per day (see [5]). Similar recommendations were subsequently forthcoming from The Australian Department of Health [6] and The Canadian Paediatric Society [7]. Such is the continued growing concern pertaining to SBMU that the US Department of Health and Human Services [8] now cites its reduction as a key health improvement priority in its 10 year health promotion and disease prevention objective. However, these guidelines focus on SBMU for entertainment rather than educational purposes. It has been posited, however, that irrespective of the content or educational value of what is being viewed, the sheer amount of average daily screen time during discretionary hours outside of school may be an independent risk factor, often exhibiting a dose–response relationship with disease and unfavourable child development outcomes [2]. The growing concern for the long term impact of SBMU on health across the lifespan [9], suggests there is a need to focus on all SBMU. Furthermore, the increasing use of SBMU in regular primary school and high school classrooms during the regular school day, and for homework purposes, and for social networking need also to be taken into account if a more accurate estimate of SBMU and its consequences (positive and negative) are to be ascertained.

Data emerging from many studies not only suggest that the < 2 hours recommendation has gone unheeded, but that the degree to which young people exceed the < 2 hours may be increasing. For example, data covering 1999–2009 showed that 8 to 18 year olds in the USA increased the average amount of time they spent viewing TV content (on computers and other platforms) from 3 hours 45 minutes to 4 hours 30 minutes per day. Daily computer use and video gaming also increased over the same period from 27 minutes to 1 hour 29 minutes and from 26 minutes to 1 hour 13 minutes, respectively. Overall, approximately 7.5 hours was spent per day with TV, computers, video games, and movies [10].

Also in the USA, children aged > 8 years were reported to spend an average of 6.43 hours per day on electronic media [11]. Similarly, other studies show young people in the USA exceeded the < 2 hour recommendation for TV/video viewing, computer use, and total screen time. More boys (49.4%) than girls (45.0%) and older (12–15 years: 56.0%) than younger children (2–5 years: 35.3% and 6–11 years: 49.1%) spent > 2 hours daily on screen time [12]. In another study [13] 21% of 6–11 year olds and 26% of 12–17 year olds exceeded the < 2 hour daily time limit on TV viewing, video viewing and video game playing. Similar rates of excessive usage of screen time (i.e., TV, video games, computer games – but not “including time spent doing homework on the computer”) were reported in an earlier study [14]. Specifically, 27% of 9–15 year olds exceeded 2 hours, which the authors argued was within the range of results from other national surveys i.e., 32% of 6–11 year olds and 35% of 12–15 years olds > 2 hours [15]; and 35% watching TV > 2 hours on an average school day [16]. As with many other studies researchers found that across weekdays 13–15 year olds spent most time watching TV, followed by computer use and video gaming [see 1].

Parent reports about the amount of time their child “usually uses a computer for purposes other than schoolwork” and “watches TV, watches videos or plays computer games” on a typical weekday, have also revealed that 49% of 6–17 year olds in the USA exceeded 2 hours per day and 16% exceeded > 4 hours per day [17].

In Australia, 58.9% and 75% of children respectively, exceeded the < 2 hours per day recommendation for SBMU [18, 19]; higher rates exceeding the recommended guidelines (80% and 87%) were subsequently reported in two other studies [20, 21], respectively. In a recent Australian study the authors did not comment on whether the participants exceeded the < 2 hour time recommendation, but data from 643 14 year olds revealed an overall median SBMU time per week of 30.1 hours for males and 21.2 hours for females [22].

In the UK, 60% to 70% of 15 year olds watch TV > 2 hours per day [23], while the proportion of young people exceeding 2 hours playing computer games has increased from 42% to 55% (boys) and 14% to 20% (girls) from 2006 to 2010 [24]. Similar levels exceeding < 2 hours SBMU per day have also been reported in Norway [25–27], but in the latter study only a single item was used [27]. Spanish studies also provide further evidence of cross cultural homogeneity concerning this issue: 35.2% and 32.3% of boys and girls aged 15–18 years viewed TV for > 2 hours per weekday; 21.6% of boys and 9% of girls played computer games for at least 2 hours per weekday; and 7.6% of boys and 0.5% of girls exceeded recommended limits on videogames [28]. Furthermore, overall screen use (TV, mobile phone, computer/videogames) has been reported to be 2.52 hours per day [29], and 50% of 14–18 year olds exceeded the < 2 hours per day recommendation for TV viewing and approximately 66% for computer use [9].

Thus, there is a clear international evidence base which indicates that young people’s SBMU exceeds the < 2 hours recommendation and that for entertainment purposes such electronic media are adversely related to health and developmental outcomes [2]. However, there are now plausible arguments that excessive SBMU, including for educational purposes, might have adverse effects on adolescent health and that the educational value of the SBMU does not preclude the significant associations between SBMU and morbidity and mortality [2]. For example, children using screens for an average of three hours per day have been found to experience a stress response (i.e., reduced cortisol increase) on waking the following day, which suggests the development of an allostatic load from repeated exposure to screens; in turn this may predispose the child to accelerated pathophysiology and unfavourable health outcomes in later years [see 2 for a review].

The use of mobile devices now makes screen use the centrepiece of young people’s social lives [30]. Children and adolescents live in media saturated worlds [31] where the introduction of newer mobile screen media has afforded them with unprecedented access to the wider world and hence a variety of activities (e.g., video gaming, social networking, internet searching, TV watching) for academic, social and entertainment purposes. That these devices have been “embraced by younger generations more quickly and incorporated more seamlessly into their daily routines has heightened concerns” [32], p. 2. Thus, the viability of achieving < 2 hours per day of SBMU may be difficult.

In summary, substantial numbers of young people exceed the recommended < 2 hours per day for SBMU, irrespective of country of residence. Given young people’s lifestyles are set in a world comprising a range of electronic devices that are integrated seamlessly into their daily routines, should this come as any surprise? Almost all studies to date have focused solely on estimates based on young people’s use of a single screen (e.g., TV watching) and have used that as a proxy for SBMU more generally rather than asking directly about multiple SBMU. Consequently, the true extent of SBMU may remain relatively unknown. While gaining data on overall SBMU as a single construct is important, it is also essential to determine the different types of SBMU being utilised and for what activities, because there is evidence of their differential effects on health (see [33]). Furthermore, although there is some evidence of sex and age differences in SBMU the full extent of this across the child and adolescent age range remains relatively unknown.

### Hypotheses

Our main research questions relate to the degree to which young people report exceeding the SBMU recommendations, first according to overall SBMU estimates and then according to each of four broad activity types. We are also interested in the extent to which sex and school-stage (Grade level) are associated with excessive SBMU. Based on our literature review, we expected that more boys than girls would exceed the recommended SBMU guidelines of < 2 hours per day and that older participants would be more likely to exceed the guidelines than younger participants. With regards to specific forms of SBMU, we expected that boys would be more likely to exceed guidelines based upon levels of gaming alone than girls would be. We also expected girls would be more likely than boys to break the guidelines regarding social networking given recent evidence suggesting that adolescent girls spend more time on such activities than do adolescent boys [10].

## Methods

### Participants and settings

The data presented from this cross sectional online survey was obtained from a total of 2,620 children and adolescents (1,373 males and 1,247 females) from Grade 3 (8 years of age: 301 males, 303 females), Grade 5 (10 years of age: 346 males, 307 females), Grade 7 (12/13 years of age: 370 males, 324 females), and Grade 9 (15/16 years of age: 356 males, 312 females) from 25 randomly selected schools. Of the schools, 14 were state government primary schools (4 rural locations), 6 were state government high schools (3 rural locations), 1 was a state government District high school (rural location catering for grades K to 10) and 4 were non-government schools (K – 12).

All participating schools were located across different socio-economic status (SES) areas as indexed by their Socio-Economic Index for Areas (SEIFA), Australia, 2011 [34]. Seven primary schools were in low SES areas, four in mid SES areas, and three were in high SES areas. Of the six state government high schools three were in low SES areas and three were in mid SES areas. The District High school was in a low SES area and of the four non-government high schools three were in high SES areas and one was in a mid SES area.

In conducting the research we adhered to the STROBE (STrengthening the Reporting of OBservational studies in Epidemiology) statement for cross sectional and observational studies.

### Instrumentation

The Screen Based Media Use Scale (SBMUS) was specifically developed to measure daily SBMU. Initially, the instruments and/or items used by researchers in previous research were reviewed to identify items for possible inclusion in the SBMUS. It was evident that most studies relied on either one item or two items to collect data on “total time spent on screens” and that these data related to TV watching, *or* computer use, *or* video game playing, or a combination of these. We sought information from young people about the different types of screens being utilised, by whom (i.e., according to sex and age) and for what purpose. Therefore, our instrument comprised the following sections and format: (i) Demographics; A brief section seeking information about sex, date of birth, age, and school grade level. (ii) Screen types; the following text was prominently displayed “*Screens can mean anything that shows a picture that you watch or interact with. Below are some pictures of screens you may use. These include an iPod Touch, iPad, Mobile Phone or iPhone, TV, Laptop Computer, Portable PlayStation or an Xbox.* (Images of these screens were then presented.) *Examples of things you can do on screens are watch TV, search the internet, use social networking sites, use instant messenger, send and receive emails, play games, online shopping, download music, do school work and homework, and watch music videos”.* The images of the eight screen based media were then presented again and participants were requested to place a check in the box of any that they had used in the last seven days.

An interactive slide bar that measured SBMU in hours and minutes was then presented and participants were asked to “*think about****ONE****typical day last week (Monday to Friday). How many****HOURS****in total did you spend on****ALL****screens that DAY? Start from the time****you woke up****and think about the****total number of hours****, including before school, during school, after school, at home or at a friend’s house, and in the evening until the time****you went to bed****.* (Emphases shown are as displayed on the online SBMUS.) Employing a sub-sample of 174 young people, we were able to assess test-retest reliability of this measure across a six month period. Overall reliability was good (*r* = .50, *N* = 174) and this did not differ by gender (*r*_*boys*_ = .51, *n* = 91; *r*_*girls*_ = .53, *n* = 82). Reliability varied somewhat across Grades 3 (*r* = .49, *n* = 33), 5 (*r* = .60, *n* = 44), and 7 (*r* = .52, *n* = 51). However, test-retest reliability was most problematic amongst the oldest group, those in Grade 9 (*r* = .19, *n* = 46). However, the young people in Grade 9 were taking examinations during the second point in time and we believe that this disrupted the stability of the measure.

(iii) Screen activities; A list of 20 screen based activities (e.g., used Google, Twitter, played MMORPG, online shopping, used the web for research, watched/listened to videos/music) along with illustrated images were then presented and participants were asked to place a check in the box of any they had participated in over the past seven days.

Four separate sections (each with definitions and an illustrated image of what the section referred to) on gaming, social networking and instant messenger, TV/Videos/Music, and searching the web were then presented. Each of the four sections required participants to use an interactive slide bar to estimate their SBMU in hours and minutes [as in (i) above]. The same sub-sample of young people provided test-retest data for each of these four measures. Overall, test-retest reliabilities were good, ranging from .46 (Web use) to .53 (Gaming) for the sub-sample as a whole (*N* = 174). Test-retest reliability was higher for girls on Gaming (*r*_*girls*_ = .65, *n* = 68; *r*_*boys*_ = .49, *n* = 78), Social networking (*r*_*girls*_ = .74, *n* = 77; *r*_*boys*_ = .22, *n* = 84) and Web use (*r*_*girls*_ = .52, *n* = 77; *r*_*boys*_ = .35, *n* = 87), but was higher for boys on TV/Videos/Music (*r*_*boys*_ = .58, *n* = 89; *r*_*girls*_ = .53, *n* = 77). Across Grades 5 and 7, test-retest figures for all four measures ranged from .51 to .69. At Grade 3, reliabilities were .41 or .42 except for Social networking which was very poor (*r* = .08, *n* = 26). At Grade 9, reliabilities were also good for three of the measures (*r*_*gaming*_ = .50, *n* = 37; *r*_*social networking*_ = .80, *n* = 45; *r*_*tv*_ = .53, *n* = 45) but the fourth, Web use, was weaker (*r* = .22, *n* = 45). This may also reflect the same issue as noted previously, that the oldest children were engaged in exams during the later data collection and Web use is likely to be one of the primary uses of screen when at school. This approach allowed an overall time estimate and then a time estimate of each of the four activity types. This also meant that we could consider the prevalence of adolescents exceeding the < 2 hours recommendation and to what degree.

To test the instrument 20 young people aged 8–16 years were provided with unique log in codes and asked to comment on face and content appropriateness of the instrument, its ease of use, interactivity and engagement. With the exception of a slight modification to the interactive slide bars (modified to show amount of SBMU time in numbers as the bars slide) to estimate the amount of time spent using screen based media, all feedback was positive. On average participants completed the SBMUS in 25 minutes.

### Procedure

Permission to conduct the research was initially obtained from the Human Research Ethics Committees of the University of Western Australia and the State Department of Education. Following this 30 schools were randomly selected from a mix of socioeconomic and metropolitan/rural areas and their Principals were contacted to ascertain their interest in participating. The 25 who expressed an interest subsequently received information sheets explaining the research, along with a follow up phone call to answer any questions and to finalise their involvement. As this current research is part of a three year longitudinal study using an accelerated cohort sequential design, information sheets and consent forms (for active informed consent from parents) were sent to the parents of children in primary school Grades 3, 5, and 7 and adolescents in high school Grade 9. The sample of 2,620 students represented an affirmative return rate of approximately 78%.

The SBMUS was administered to the participants in groups of 15–25 students during a four week period when the electronic link remained open. All participants were provided with a unique code that allowed them to access the online version of the SBMUS to complete in confidence. Administration in each school was supervised by a member of staff who received a written set of instructions to ensure standardization of administration and to address any technical difficulties should they arise.

### Statistical analyses

Our analyses included four sections. The first section included descriptive data. In the second section, our aim was to examine the possible association of Sex (male, female) and Grade (3, 5, 7, 9) with participants’ overall assessment of their weekday SBMU (<2 hours, > 2 hours). We achieved this by conducting a hierarchical linear model, using backwards elimination of effects. Where there were significant effects, these were interrogated by calculating relative odds ratios (Field, 2009).

In the third section, our aim was to investigate the data in more detail. The same hierarchical linear model was used but we replaced ‘overall assessment of SBMU’ with the estimated screen time for each of four different forms of SBMU: Gaming, Social Networking, Web use, and TV/DVD/Movies. Thus, for example, the first hierarchical linear model included Sex, Grade (3, 5, 7, 9), and participants’ assessment of their weekday SBMU for Gaming (<2 hours, > 2 hours).

In the fourth and final section, our goal was to examine the extent to which young people reported exceeding SBMU recommendations on different forms of screen use (Gaming, Social Networking, Web use, and TV/DVD/Movies). To this end, we created a variable which reflected the number of screen activities which participants reported engaging in on weekdays for more than 2 hours. This measure could range from 0 (no screen activities exceeding 2 hours) to 4 (use of all four screen activities exceeded 2 hours). We then examined whether this variable differed as a consequence of Sex and Grade (3, 5, 7, 9) by conducting a two-way independent ANOVA. A significant interaction was interrogated by conducting four independent *t*-tests comparing boys’ and girls’ scores at each grade level. These *t*-tests were Bonferroni corrected (.05/4 = .013).

### Ethical standards

The authors assert that all procedures contributing to this work comply with the ethical standards of the relevant national and institutional committees on human experimentation, the American Psychological Association and with the Helsinki Declaration of 1975, as revised in 2008.

## Results

### Descriptive data

The data collected included information relating to the specific ways in which young people reported engaging in SBMU. Here, the young people were asked to say whether they did or did not engage in each of 20 different screen based activities. As shown in Table 1, the most popular activity was watching TV (94%) and listening to music or watching videos (92%). The least reported activities included Twitter (8%), MMORGs (21%) and online shopping (21%).

**Table 1:** **Numbers (and proportions) of young people reporting engaging in specific forms of screen use**

Activity	Number engaging in activity (%)
Watched TV	2381 (93.7%)
Listen Music, Watch Videos	2310 (91.8%)
Google	2176 (86.0%)
Research, web Use for School Work	1569 (63.6%)
Download Music Videos or Music	1446 (57.8%)
Email	1335 (54.7%)
Indie Games	1050 (42.9%)
Puzzle Board Card Games	1005 (40.9%)
Kik/Tumblr/Instagram	997 (40.5%)
Action Adventure Games	930 (37.7%)
Shooter Games	891 (36.2%)
Driving Games	873 (35.8%)
Sports Games	842 (34.8%)
Facebook	852 (33.9%)
Instant Messenger	658 (26.9%)
Rhythm/Music Games	562 (23.1%)
Role Playing Games	563 (23.1%)
MMORG	519 (21.1%)
Online Shopping	498 (20.6%)
Twitter	118 (7.8%)

While the main analyses below related to the presence or absence of problematic SBMU, we also present the means and standard deviations for the different forms of SBMU (see Table 2). Given the degree to which these variables were skewed, we did not conduct ANOVAs to look for effects of sex and grade, instead leaving investigation of these effects until our analyses below.

**Table 2:** **N (and percentage) of students engaging in screen use for more than two hours per weekday, by sex, grade, and screen activity**

Sex	Grade	Gaming	Social networking	Web	TV/DVD/Movie	Global ^a^
Male	3	2.63 (2.88)	0.82 (1.97)	1.74 (2.43)	2.63 (2.71)	3.16 (3.04)
	5	2.02 (2.02)	0.77 (1.61)	1.16 (1.59)	2.10 (2.01)	2.76 (2.38)
	7	1.86 (2.09)	1.20 (2.10)	1.32 (1.82)	2.17 (2.20)	3.02 (2.32)
	9	1.86 (2.56)	1.96 (2.87)	1.67 (1.96)	2.52 (2.45)	3.81 (2.59)
Female	3	1.98 (2.54)	0.47 (1.47)	1.50 (2.23)	2.50 (2.43)	2.69 (2.46)
	5	1.84 (2.52)	1.07 (1.96)	1.73 (2.37)	2.68 (2.64)	3.10 (2.59)
	7	1.41 (2.06)	2.13 (2.70)	2.11 (2.27)	3.02 (2.61)	4.61 (2.64)
	9	1.07 (2.02)	3.18 (3.33)	2.54 (2.53)	3.34 (2.83)	6.13 (2.84)

^a^Global = Overall assessment of time spent interacting with screens per week day, not a cumulative total of different activities.

The durations to which children and adolescents reported SBMU during a typical weekday, in the light of AAP (and Australian Department of Health) recommendations is presented. This was investigated first for their general estimate of daily SBMU, and subsequently for SBMU pertaining to each group of screen based activities.

### Overall screen use: within AAP recommendations (≤2 hours) or in excess (>2 hours)

The number and percentage of participants engaging in SBMU for > 2 hours on a typical weekday, by Sex, Grade, and Screen Activity, is shown in Table 3. On a typical weekday, 62.7% of participants exceeded the < 2 hours recommendation. Approximately 45% of the youngest participants (i.e., Year 3, age 8 years) exceeded the < 2 hours recommendation, rising to 80% by Year 9 (15–16 years of age). The most popular screen of use was TV, with almost 90% reporting watching TV in the week prior to the survey. This was followed by laptop (59%), iPad/Tablet (58%) and mobile phone (57%) use. A three-way frequency analysis was performed to develop a hierarchical linear model of Sex, Grade (3, 5, 7, 9), and overall SBMU (<2 hours, > 2 hours). Backward elimination produced a model that included only the three-way interaction effect, *χ*^*2*^ (3, *N* = 2515) = 56.18, *p* < .001. At Grade 3, there was no relationship between Sex and SBMU. At Grade 5, the relative odds ratio (Field, 2009) indicated that girls were 1.42 times more likely than boys to be in the > 2 hours group. At Grade 7, this trend was accentuated, with girls 3.71 times more likely to be in the > 2 hours group. Finally, this trend developed further in Grade 9 with girls 4.95 times more likely to be in the > 2 hours group than boys. The increasing sex disparity was evident despite the overall trend for increasing SBMU across both boys and girls (see Table 1).

**Table 3:** **N (and percentage) of students engaging in screen use for more than two hours per weekday, by sex, age, and screen activity**

Sex	Grade	Screen activity engaged in for greater than two hours each weekday				
		Gaming	Social networking	Web	TV/DVD/Movie	Global ^a^
Male	3	107 (39.8%)	30 (11.4%)	60 (23.5%)	104 (38.8%)	132 (46.5%)
	5	106 (33.2%)	31 (10.6%)	44 (13.9%)	104 (33.5%)	151 (45.3%)
	7	114 (33.4%)	57 (16.8%)	57 (16.4%)	131 (37.5%)	201 (55.5%)
	9	84 (26.8%)	83 (25.3%)	71 (21.9%)	139 (41.9%)	237 (69.5%)
Female	3	79 (30.0%)	15 (5.6%)	58 (21.5%)	102 (38.1%)	124 (43.4%)
	5	63 (22.4%)	47 (17.7%)	57 (20.7%)	119 (41.9%)	161 (54.2%)
	7	61 (20.8%)	100 (32.3%)	101 (32.6%)	160 (51.1%)	260 (82.3%)
	9	42 (15.6%)	141 (48.5%)	118 (40.1%)	162 (54.9%)	271 (91.9%)

^a^Global = Overall assessment of time spent interacting with screens per week day, not a cumulative total of different activities.

### Specific screen use group activities: within AAP recommendations (<2 hours) or in excess (>2 hours)

Next, we examined the extent to which each form of SBMU exceeded the recommendation of < 2 hours. To achieve this, we conducted four further hierarchical linear models, each with Sex, Grade (3, 5, 7, 9), and specific form of SBMU (<2 hours, > 2 hours) included.

For *Gaming*, the final model had a likelihood ratio *χ*^*2*^ (6, *N* = 2349) = 4.35, *p* = .629, indicating a good fit between the observed and predicted frequencies. There was no interaction between Sex and Grade, but there was a main effect of Sex: boys were 1.75 times more likely than girls to exceed the < 2 hour recommendation for gaming. There was also a main effect of Grade: Grade 3 children were approximately 1.4 times more likely than Grades 5 and 7 (relative odds 1.38 and 1.42 respectively), and 1.93 times more likely than Grade 9, to exceed the < 2 hour recommendation for gaming. Grades 5 and 7 did not differ from each other, and both these grades were more likely than Grade 9 (relative odds 1.39 and 1.36 respectively) to exceed the < 2 hour recommendation (see Table 3).

For *Social Networking*, the best fitting model included only the three-way interaction effect, *χ*^*2*^ (3, *N* = 2357) = 25.85, *p* < .001. At Grade 3, boys were 2.17 times more likely than girls to exceed the < 2 hour recommendation using social networking. However, at Grade 5 girls were 1.75 times more likely to exceed the < 2 hour recommendation using social networking, and this increased at Grade 7 (2.40 times more likely than boys) and again at Grade 9 (2.76 times more likely than boys). So, as was the case for overall SBMU, social networking increased with age, but increased much more rapidly for girls. Specifically, Grade 9 girls were 15.67 times more likely to exceed the < 2 hour recommendation using social networking when compared to Grade 3 girls, while the equivalent statistic for boys was 2.61 (see Table 3).

For *Web Use*, the best fitting model again included only the three-way interaction effect, *χ*^*2*^ (3, *N* = 2395) = 16.80, *p* = .001. At Grade 3, there was no association between Sex and SBMU relating to the Web. However, by Grade 5, girls were 1.63 times more likely to exceed the < 2 hour recommendation using the Web, and this increased at Grade 7 (2.40 times more likely than boys) and then remained at that level at Grade 9 (2.39 times more likely than boys). These effects seem to be due to a dip in boys’ Web use at Grade 5 before returning to the same level again by Grade 9, whereas girls’ Web use stayed the same at Grade 5 but increased thereafter (see Table 3).

With respect to TV/DVD/Movies, the final model had a likelihood ratio *χ*^*2*^ (6, *N* = 2419) = 9.365, *p* = .154. There was no interaction between Sex and Grade, but there was a main effect of Sex: girls were 1.44 times more likely than were boys to exceed the < 2 hour recommendation using TV/DVD/Movies. There was also a main effect of Grade. Grades 3 and 5 did not differ in their SBMU for this purpose, but Grade 7 were more likely than both Grades 3 and 5 (relative odds 1.24 and 1.30 respectively) to exceed the < 2 hour recommendation using TV/DVD/Movies. Grade 9 were slightly more likely to use screens for watching TV/DVD/Movies than were Grade 7 (relative odds of 1.18), but were more noticeably different from Grades 3 and 5 (relative odds 1.46 and 1.53 respectively) (see Table 3).

### Multiple screen use: within AAP recommendations (<2 hours) or in excess (>2 hours)

Finally, in recognition of the fact that children and young people multi-task in their SBMU, we investigated the degree to which young people reported exceeding SBMU recommendations on more than one activity (Social Networking, Gaming, Web Use, TV/DVD/Movies). Our data indicated that 4.3% exceeded the < 2 hour recommendation on all four separate screen activities, 10.0% on three, 16.6% on two, and 24.6% on one. Under half (44.5%) did not exceed the < 2 hour recommendations on any of the four screen activities. This is higher than the single-item global estimate of SBMU reported above, which indicated that 37.3% of young people did not exceed the < 2 hour recommendations. Using this measure (number of screen activities where the < 2 hours recommendation is exceeded) as a dependent variable, we conducted a two-way independent ANOVA, with Sex (male, female) and Grade (Grades 3, 5, 7 and 9) as the independent variables. This revealed a small interaction between Sex and Grade: *F* (3, 2589) = 8.32, *p* < .001, *η*_*p*_^*2*^ = .01. Boys in Grade 3 reported exceeding the < 2 hours recommendation on a greater number of activities than girls in Grade 3. By Grade 5, girls’ scores on this measure marginally exceeded those of boys and in Grades 7 and 9 girls’ scores clearly exceeded boys’ (see Table 4).

**Table 4:** **Mean number of screen activities where the < 2 hours recommendation is broken, by sex and grade**

Sex	Grade				Total
	3	5	7	9	
Male	1.02 (1.22)_a_	0.82 (1.06)_a_	0.97 (1.17)_a_	1.08 (1.16)_a_	0.97 (1.15)
Female	0.84 (1.11)_a_	0.93 (1.20)_a_	1.30 (1.21)_b_	1.51 (1.20)_b_	1.15 (1.20)
Total	0.93 (1.17)	0.88 (1.13)	1.13 (1.20)	1.28 (1.18)	1.06 (1.18)

*Note*: where subscripts differ in columns, boys and girls differed significantly (*p* < .013).

## Discussion

This research appears to be among the first to examine all types of children’s and adolescents’ SBMU, rather than as in most studies conducted to date, solely TV or computer videogame playing (or a combination of both) (see [35]). Although previous Australian research [21] presented a list of eight screens to their participants, there appears to have been limited reference to newer mobile devices. Furthermore, the age range of the sample was limited (11–12 year olds). The online instrument we developed utilised multiple items to gather data on SBMU on eight separate screen media and the amount of time participants spent on four activities (gaming, social networking, TV/Videos/Music, and searching the Web) on these devices. This approach is preferable, as treating SBMU only as a single construct may result in important information being missed [36] or that the unique effects of any one medium will be difficult to identify [35]. Moreover, separating according to screen only (e.g., TV, computer use) may not be sufficiently specific to understand any relationships between SBMU activities (see [22]).

The arrival of new mobile screen media provides young people with access to an increased variety of activities and at any time of their choosing. Thus, if accurate estimates of SBMU are to be obtained then not only should all SBMU be investigated, but so too should their usage throughout the waking day, including school time. To address this we gathered data on SBMU from the time participants woke until the time they went to bed, including for academic and non-academic related activities during regular school hours. If government departments are serious about making evidence based policies regarding the effects of SBMU on physical and mental health, then determining what young people are using and when, is the first step to understanding whether specific SBMU is associated with distinct positive or negative health risks (see [22]).

The evidence to date is unequivocal that significant proportions of young people the world over are exceeding the < 2 hours per day recommendation [4]. This present study provides supporting evidence that excessive SBMU is also common among Western Australian children and adolescents. Our global measure of screen use indicated that 63% of young people exceeded the recommendation of < 2 hours per day, while our measure examining use of screens across four specific forms of screen use suggests that 55% exceed the recommendation. Although the prevalence rate among Western Australian children and adolescents is greater than that shown previously in many USA based studies, many of these (USA) studies tended to focus on a limited range of SBMU (TV, and/or computer) and did not include time spent doing schoolwork or homework. Therefore, it is not surprising that reported prevalence rates were lower. Nonetheless, similar SBMU rates to the present study have been reported among adolescents in USA studies e.g., [17], even though information was only sought about TV, videos and video games, and “computer use for purposes other than schoolwork”. Previous studies in the UK [23], Spain [9, 29], Norway [25–27], and Iceland [3] also found excessive prevalence rates of SBMU.

In comparison to other Australian studies reporting the proportion of young people exceeding the < 2 hour recommendation, the present findings are comparable to two studies [18, 19], but substantially lower than two others [20, 21]. A smaller sample with a limited age range (11–12 years) may be one reason for the higher prevalence rates reported in one of the studies [21]. In addition, the time spent on different types of electronic media were summed to produce an overall time estimate [21] and given young people’s periods of multiple device use this may have produced an inflated outcome. With reference to the other study with substantially different findings [20] the participants were asked on how many days (of the past seven) had they “watched television, played electronic games, or used the computer (other than for homework) for a combined total of less than 2 hours per day” [20]. Those who responded seven days were classified as meeting guidelines, those who responded six or less were classified as not meeting guidelines (on all of the past 7 days). This may be why substantial differences are evident between the studies.

Sex and age must also be taken into consideration, since SBMU is not equally distributed across sex and school grade level (see [27]). Our model of sex, school grade level and overall SBMU (<2 hours, > 2 hours) supported our hypothesis that SBMU would be higher among older participants: with the exception of Grade 3 there was an overall trend for increasing SBMU across both boys and girls as they got older (up to 16 years). This reflects previous research showing that older children are more likely than younger ones to spend more time on screen activities (see [37–39]). Contrary to our hypothesis, girls in the present study were more likely than boys to exceed the < 2 hours per day recommendation, and this difference increased with increasing school grade level. These sex and age findings may reflect the increasing use of SBMU in later grade primary school classes and high school classes, and also for homework purposes, the latter being more frequent with increasing age [38]. Although consistent with research showing significant differences in SBMU patterns according to sex [31], these current findings are generally inconsistent with studies that suggest boys spend more time than girls on screen activities e.g., [21, 27, 38, 39]. However, this needs further clarification because SBMU is not homogenous given the different stationary and mobile devices available [1]. Similarly, it has been posited that researchers should examine SBMU according to specific screen time activities [22].

The models we constructed for sex, school grade and specific form of SBMU activity (<2 hours, > 2 hours) revealed different patterns of SBMU. As hypothesised, it was only in *Gaming* that boys were more likely than girls to exceed the < 2 hours recommendation*.* That boys spend more time in total gaming is well established e.g., [21, 40, 41] and the present research findings add to this. However, the present findings also add to the limited research evidence that greater proportions of boys than girls exceed the daily < 2 hours recommendation for gaming (see [3, 24, 28, 42]). Generally, as they get older the likelihood of exceeding the < 2 hours recommendation for gaming decreases, however. This may be a reflection of indulgence in other forms of newer media (e.g., cell phones, iPads) on which gaming is only one activity among many on offer (e.g., social networking, film and TV viewing). These other activities may act as a substitute for gaming because during adolescence a range of media become especially important as young people seek to establish their identities. In doing so they spend increasing amounts of time with their peers, use newer technologies to develop niche interests [31], and have greater levels of unsupervised time with newer screen based media devices (see [43]).

For the other SBMU activities (i.e., *Social Networking, Web use*, and *TV/DVD/Movies*) the opposite was true, however, with girls more likely than boys to exceed the < 2 hours recommendation. While we expected to see such a pattern for Social Networking, we had not anticipated this sex split across the other SBMU, and they contradict research [3] showing that, with the exception of internet chat channels, boys engaged in more use of all types of screens than girls. Furthermore, Grade 5 (age 10) appears to be the age at which the likelihood of girls exceeding the < 2 hours recommendation becomes critical. Other studies have also reported that girls spend more time on Social Networking Sites (SNS) (see [10, 44, 45]) and on texting and instant messaging [45] than boys. Of particular interest in the present study is the rate at which girls are more likely to exceed the < 2 hours recommendation for *Social Networking* as they got older. Specifically, by 15 years of age girls were over 15 times more likely to exceed the < 2 hours recommendation compared to their Grade 3 peers, and almost 7 times more so than boys. Although research points to improved positive psychological health and wellbeing [46] and emotional support [47] from SNS use, excessive time online also encourages social isolation and limits social support network development, thereby adversely affecting mental wellbeing. Indeed, there is evidence that SNS can increase risk for depression, anxiety and other internalizing problems, especially when social integration is not achieved [48, 49]. An association has also been found between TV viewing and depressive symptoms among adolescent girls [50]. Thus, girls may be particularly at risk for mental health problems, hence heightening the need to understand sex specific relations between SBMU and mental health and psychological wellbeing.

Research has also drawn attention to the potential benefits of certain SBMU activities (see [21]). Furthermore, specific SBMU activities such as web use are important elements of everyday school - and - homework based activities. The inherent belief that the < 2 hours recommendation for overall SBMU should be applied to all SBMU because of potential adverse outcomes may therefore be antithetical to current research findings. Indeed, should all SBMU activities be discouraged if they do not adversely impact on young people’s health related behaviours? Research examining the complex relationships between SBMU (and SNS in particular) and young people’s wellbeing is scarce, while research involving SNS and child and adolescent health is in its infancy [51].

Research has drawn attention to the importance of obtaining estimates of distinct types of SBMU if the independent relationships between them and health status are to be understood [52]. While we have not investigated relationships between SBMU and health status in the current study, the disparity between young people’s global estimates of time spent using screens during a typical weekday and their estimates based on specific screen based activities indicate to us that this measurement issue is one which may have genuine implications for research and policy. Whether requiring children and young people to simultaneously consider how long they spend on a number of screen activities leads to an over-estimate of screen time, or whether investigating discrete screen activities leads to an under-estimate of SBMU cannot be answered using our current data. Future studies should seek to address this issue by incorporating objective measures of screen use. One such example is Curbi [53], an iPhone app specifically developed to assist parents and schools to monitor and manage the SBMU of young people. Curbi provides real time empirical evidence through the 24 hour period pertaining to what devices have been used and for what duration of time, and for example, what sites have been visited.

Our current findings add additional concern to that already expressed because just over 30% of the children and adolescents in this study broke the < 2 hour recommendation on two or more separate SBMU activities. In addition, although not the case for boys, Grades 7 and 9 girls broke the recommendation on a greater number of activities than Grade 3 and 5 girls, because of their more marked use of SNS, WEB and TV as they get older.

This study has some limitations. Our findings are based on self-report. However, there is evidence that self-report measures are an effective means of obtaining an accurate insight into the subjective dispositions that can be difficult to obtain from third parties such as teachers and parents (see [54–56]). Moreover, there have been concerns regarding limited parental awareness of their child’s SBMU, along with inaccurate estimates of SBMU due to inconsistencies of what counts as SBMU [17].

It must also be acknowledged that current guidelines recommending < 2 hours of SBMU per day focus on SBMU for entertainment purposes only. The instrument developed in the present study did not separate educational and non-educational SBMU when assessing compliance with the recommended guidelines and this may potentially have deflated estimates of compliance. Furthermore, it should be recognised that the new generation screen based media used by young people does not necessarily mean that any associated behaviour is sedentary during time of use. Our instrument did not investigate this, nor did it take into account the context of use. These issues should be a focus of future research.

Finally, while the test-retest reliability of the measure across a six month period was good for males and females and Grades 3, 5 and 7 there was an issue among the older Grade 9 participants. At the time of the retest school examinations and excursions were taking place and this may have disrupted the stability of the measure. This will be further examined in our subsequent accelerated cohort survey administrations.

Despite these limitations the research also has strengths. For example, the study generated a large sample from over 25 schools and is sufficiently large to detect excess SBMU on all selected screens, and separately for boys and girls. A wide range of SBMU devices and activities were assessed using multiple items, something that numerous previous research studies have called for. An interactive online format was also developed and piloted with children and adolescents prior to the survey so as to engage participants.

## Conclusions

In conclusion SBMU plays a pertinent and relevant role in the everyday lives of young people, and both parents and schools are enthusiastically embracing the digital age. Consequently, the < 2 hours per day recommendation [4] may no longer be tenable given the surge in social media engagement and school derived SBMU. This seems plausible across a wide range of countries not just in Australia, as reported here. Further research is now required to develop evidence based SBMU guidelines for children and adolescents in relation to the mental, social and physical health impact of such behaviours. Furthermore, as researchers, educators and health related professionals seek to develop such guidelines for appropriate SBMU, they would do well to take cognizance of the extent to which screen use differs across form, activity, sex, and age.

## Authors’ information

SH is a registered psychologist and Director of the Centre for Child & Adolescent Related Disorders, The University of Western Australia. He is also Visiting Professor School of Psychological Sciences and Health, University of Strathclyde, Glasgow, Scotland. SCH is a Developmental Psychologist, at the School of Psychological Sciences and Health, University of Strathclyde, Glasgow, Scotland. MR is Director of the Health Promotion Evaluation Unit at The University of Western Australia. LW is an Associate Professor in the School of Population Health, The University of Western Australia and held a Senior Research Fellowship from Healthway. CZ is in the Graduate School of Education, The University of Western Australia, has a background in health promotion and is the research officer and manager of the program from which this research emanates. KM is an Assistant Professor in the School of Population Health, The University of Western Australia and holds a Research Fellowship from Healthway. Trevor Shilton is Director of Cardiovascular Health at the National Heart Foundation and is Adjunct Professor at The University of Western Australia.
